# Amelogenesis Imperfecta and Screening of Mutation in Amelogenin Gene

**DOI:** 10.1155/2014/319680

**Published:** 2014-06-19

**Authors:** Fernanda Veronese Oliveira, Carla Vecchione Gurgel, Tatiana Yuriko Kobayashi, Thiago José Dionísio, Lucimara Teixeira Neves, Carlos Ferreira Santos, Maria Aparecida Andrade Moreira Machado, Thais Marchini Oliveira

**Affiliations:** ^1^Department of Pediatric Dentistry, Orthodontics and Community Health, Bauru School of Dentistry, University of São Paulo, Alameda Octávio Pinheiro Brisolla, No. 9-75, 17012-901 Bauru, SP, Brazil; ^2^Department of Biological Sciences, Bauru School of Dentistry, University of São Paulo, Alameda Octávio Pinheiro Brisolla, No. 9-75, 17012-901 Bauru, SP, Brazil; ^3^Hospital for the Rehabilitation of Craniofacial Anomalies, University of São Paulo, Rua Silvio Marchione, No. 3-20, 17012-900 Bauru, SP, Brazil

## Abstract

The aim of this study was to report the clinical findings and the screening of mutations of amelogenin gene of a 7-year-old boy with amelogenesis imperfecta (AI). The genomic DNA was extracted from saliva of patient and his family, followed by PCR and direct DNA sequencing. The c.261C>T mutation was found in samples of mother, father, and brother, but the mutation was not found in the sequence of the patient. This mutation is a silent mutation and a single-nucleotide polymorphism (rs2106416). Thus, it is suggested that the mutation found was not related to the clinical presence of AI. Further research is necessary to examine larger number of patients and genes related to AI.

## 1. Introduction

Amelogenesis imperfecta (AI) represents a collection of inherited disorders, which are clinically heterogeneous and exhibit tooth enamel defects in the absence of systemic manifestations [1]. AI generally affects all or nearly all teeth in both the primary and permanent dentitions [2]. The predominant clinical manifestations of affected individuals are enamel hypoplasia and hypomineralization, or a combined phenotype, which is seen in most cases [3]. AI results from single-gene mutations and can be transmitted by an autosomal-dominant, autosomal-recessive, sex-linked, and sporadic inheritance patterns, as well as sporadic cases [4]. Based upon the enamel phenotype and mode of inheritance, 14 clinical subtypes of AI are recognized. The complexity of the disease pattern suggests that mutations in many different genes cause AI [5].

In the last years, a correlation between AI and some genes encoding specific enamel proteins has been suggested by molecular studies and mutational analysis. Variations in the amelogenin gene (AMELX) sequence is associated with X-linked AI, while alterations in the sequence of enamelin gene (ENAM), enamelysin gene (MMP-20), and kallekrein-4 gene (KLK-4) cause hypoplastic or hypomaturation AI with the autosomal pattern of inheritance [6]. Recently, a mutation within the DLX3 gene has been described and associated with AI hypoplastic-hypomaturation type with taurodontism [3] while the identification of a mutation in FAM83H gene was associated with autosomal dominant hypocalcified AI [7].

Clinical implications of AI vary according to subtype and its severity, but the main problems are esthetic quality low of the dentition, dental sensitivity, poor mechanical properties of the dental tissues, and loss of vertical dimension. Additionally, in some types of AI there is an increased prevalence of caries, anterior open bite, delayed eruption, tooth impaction, or associated gingival inflammation. Furthermore, nonenamel dental anomalies such as taurodontism, congenitally missing teeth, failure of eruption, root or crown resorption, root malformations, and hypercementosis are reported to be associated with AI [8]. Another aspect that cannot be underestimated is the psychological impact on patients. The patients may show low self-esteem and excessively introspective behavior, which affects their socialization and results in reduced oral health-related quality of life [6].

The aim of this study was to report the clinical findings and the screening of mutations in amelogenin gene of a 7-year-old boy with Amelogenesis imperfecta.

## 2. Case Report

**Figure 1:**
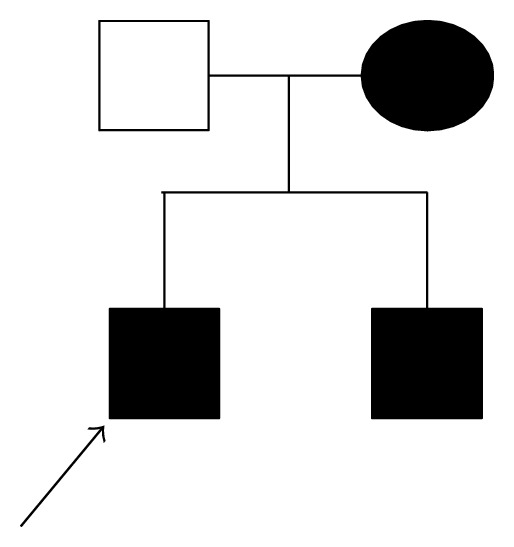
Pedigree chart of the patient's family showing the inheritance of AI in the family studied.

A 7-year-old boy was referred to the Clinic of Pediatric Dentistry of our University. His mother reported that yellowish coloration of his teeth made him ashamed of smiling and damaged his relationship with other children. A detailed dental, medical, and social history was obtained from the patient. His mother reported that she and the other son were also affected by the same dental disease (Figure 1), but their esthetic and functional rehabilitation was performed previously.

Clinical examination revealed a mixed dentition. The permanent maxillary central incisors and permanent maxillary and mandibular first molars were partially erupted. The patient exhibited poor oral hygiene with moderate to severe dental plaque accumulation and the gingival tissues around most of the permanent and primary teeth showed mild inflammation.

**Figure 2:**
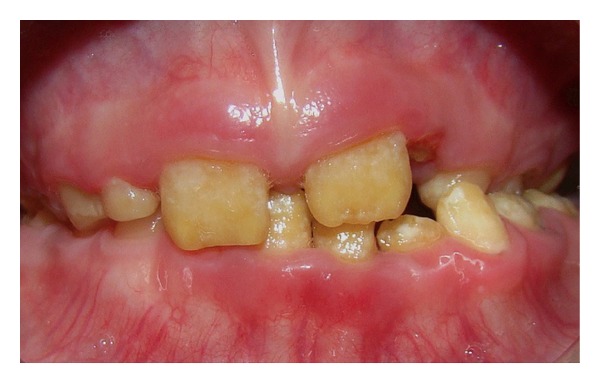
Initial intraoral view showing the teeth with AI.

**Figure 3:**
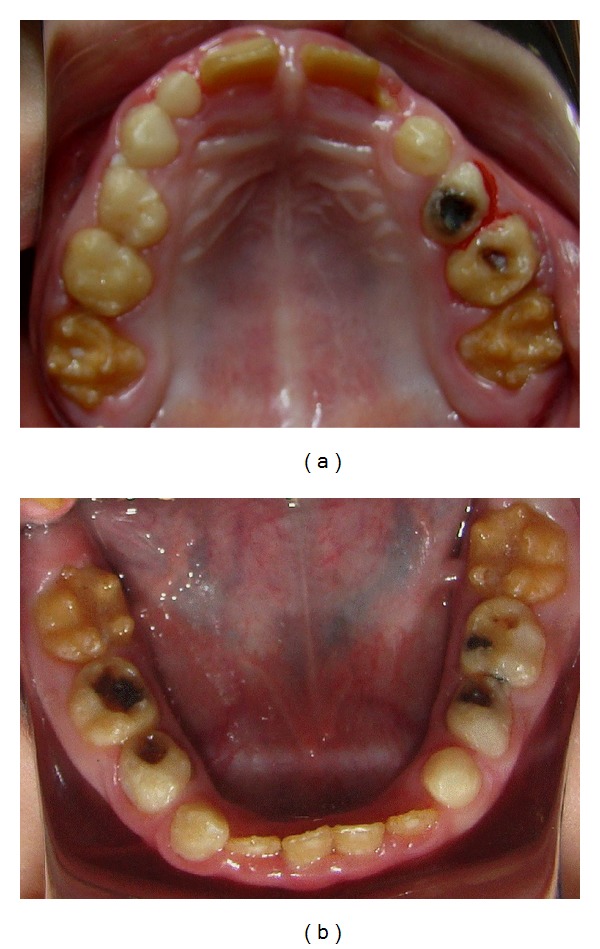
(a) Occlusal view of the maxillary; (b) occlusal view of the mandibular.

**Figure 4:**
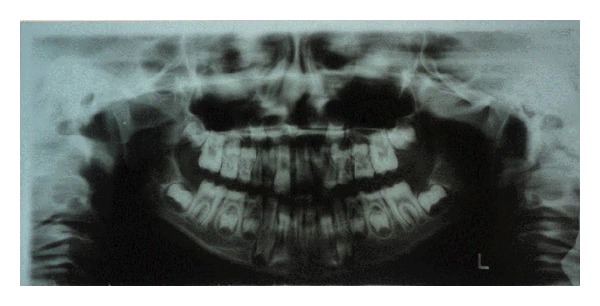
Panoramic radiograph showing that the thin enamel layer could not be distinguished from the underlying dentin.

The patient presented short clinical crowns with a generalized yellow color in both primary and permanent teeth (Figures 2, 3(a), and 3(b)). The enamel surface was thin and rough, whereas the roots showed normal length and form. The pulp chambers were regular in size. Carious lesions were present in the primary maxillary right first and second molars and in the primary mandibular first and second molars. In the right side the occlusion was in Class I and in the left side it was cross bite. A deep overbite was observed with premature loss of vertical dimension. In a panoramic radiography, the thin enamel layer could not be distinguished from the underlying dentin (Figure 4).

The treatment objectives were to improve the esthetics, eliminate the tooth sensitivity, prevent further loss of tooth structure, modify the child's attitude and behavior towards dental treatment, and improve his periodontal health. As part of the treatment plan, the treatment alternatives were explained to the child and his parents.

**Figure 5:**
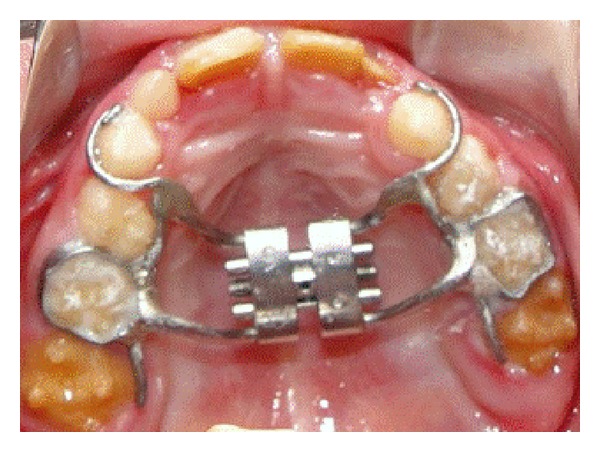
Intraoral view showing early orthodontic treatment interceptive and minimal intervention.

**Figure 6:**
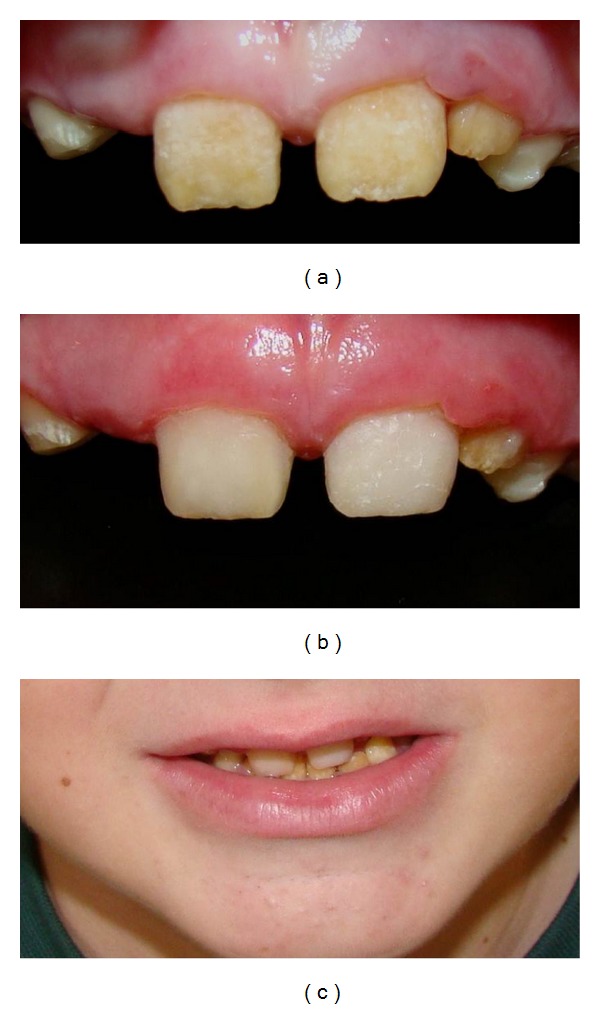
(a), (b), and (c)-Intraoral view showing the restorative treatment and esthetic appearance of the smile.

The initial treatment was early orthodontic treatment interceptive and minimal intervention. The primary maxillary right first and second molars were treated before orthodontic treatment (Figure 5). The caries-preventive strategies consisted of oral hygiene instruction and dietary recommendation. The permanent maxillary central incisors were restored with composite resin to improve esthetics. The restoration improved the esthetic appearance of the smile contributing to the improvement of his behavior and social conviviality (Figures 6(a)–6(c)). The patient presented incipient periodontal disease, and thus professional prophylaxis for dental plaque removal was carried out every three months. These recall visits were determined by patient's risk for caries/periodontal disease.

The Ethic Committee of our University approved saliva collection and mutational analysis. Saliva samples were collected from the patient, his parents, and brother for DNA analysis. The InstaGene Matrix (732-6030, Bio-Rad Laboratories, United States) protocol was used to isolate genomic DNA from saliva. All amelogenin protein coding exons sequences (exons 2, 3, 4, 5, 6, and exon 7) were amplified by Polymerase Chain Reaction (PCR) Kit (*Taq* DNA Polymerase, 11615-010, Invitrogen, Brazil).

PCR amplification products were purified by use of the QIAquick PCR Purification Kit (28106, Qiagen, Germany). The primers and PCR conditions shown in Table 1 were used to amplify the amelogenin gene.

Following purification, PCR products were sequenced using the ABI BigDye Terminator v3.1 Cycle Sequence kit (4336917, Applied Biosystems, United States) and an ABI 3130xl Genetic Analyzer (Applied Biosystems, United States). All products were sequenced from both directions to minimize sequencing artifacts. Amelogenin gene mutations were confirmed by repeating the PCR amplification and sequencing.

The electropherograms were analyzed using the SeqScape Software (Applied Biosystems, United States). The resulting sequences of direct sequencing of amelogenin gene exons were compared with the amelogenin genomic reference sequence (NC_000023.10). The variations in these sequences were checked on dbSNPs database.

Mutational analysis was performed for the coding exons of the amelogenin gene and the c.261C>T mutation was detected in samples of mother, father, and brother. This alteration is a silent mutation and a single-nucleotide polymorphism (rs2106416). However, this mutation was not found in the sequence of the patient (Table 2).

## 3. Discussion

This research used the direct sequencing method of genomic DNA from saliva samples. The fast advancements in the development of sequencing technologies, in the last years, enable an increasing number of applications in biology and several health areas [9]. The direct sequencing of genomic DNA has been the election method to investigate polymorphisms and mutations in populations and to relate them to diseases [10, 11].

According to OMIM (Online Mendelian Inheritance in Man), the amelogenin gene is localized on Xp22.31-p22.1 chromosome and is also known as AMELX, AMG, AIH1, ALGN, AMGL, and AMGX. The amelogenin encodes a member of the amelogenin family of extracellular matrix protein and has an important role in biomineralization during the tooth enamel development. Mutations in this gene cause AI linked to chromosome X. Alternative splicings result in multiple transcript variants which codify different isoforms [12].

The amelogenin gene is composed of seven exons and six introns and alternative splicings result in three different transcripts, according to Ensembl [13]. In this study, all exons participating in the process of transcription of amelogenin gene were amplified and sequenced (exons 2–7). In the first analysis, the forward primers of the exons were sequenced. The reverse primer was sequenced to confirm the mutation, when this was detected.

Wright et al. [7] conducted a study with a large AI population (71 families) and observed that an evaluation of all 6 known disease-causing AI genes and several other AI candidate genes resulted in identification of the molecular etiology in approximately 60% of affected individuals and 40% of their families. They suggest that either there are numerous mutations in the noncoding regions of the tested genes or there are additional AI-causative genes yet to be identified. Given the diverse modes of inheritance (both autosomal dominant and recessive) and the heterogeneous phenotypes in those cases where genes were not identified (hypoplastic and hypomineralized), it is probable that multiple AI genes remain to be discovered.

Although the defects caused by AI are not a public health problem, they may cause severe esthetic alterations and compromise tooth enamel structure [14]. In this case, the roughness surfaces of teeth facilitate the accumulation of dental plaque and interfere in the oral hygiene. Thus, this patient presented incipient periodontal disease. Furthermore, periodic reinforcement was conducted by professional prophylaxis for preventing caries/periodontal disease.

The severe forms may lead to early enamel loss, causing accentuated weariness with injury of the tooth organ function. It may one day be practical and highly beneficial to determine the specific AI genotype and associated AI phenotype before rendering treatment to optimize preventive and restorative care. By studying the outcomes of various restorative procedures for each genotype/phenotype condition, practicing dentists will be able to use gene-based diagnoses to select the most optimal treatment approaches and thereby restore the dentition in a way that achieves the best results [3, 15].

The results obtained from direct sequencing of protein-coding exons of the amelogenin gene may affirm that the mutation found is not related to the clinical presence of AI on individuals involved, because this mutation does not alter the amino acid or protein produced. Further research is warranted to examine larger number of patients and genes related to AI.

The exact mechanism involving tooth enamel formation is still partially obscure. Some codified genes for specific proteins of enamel have been identified by candidate genes for tooth malformations. Mutational analysis, with family study, has supported this hypothesis [3, 5, 7, 16]. On the other hand, the method of direct sequencing will enable the prediction of which candidate genes may be defective, given the tooth phenotype of the patients. This knowledge will be useful for considerate treatment alternatives. In this study, the c.261C>T mutation was found in samples of mother, father, and brother, but the mutation was not found in the sequence of the patient. This mutation is a silent mutation and a single-nucleotide polymorphism (rs2106416). Thus, it is suggested that the mutation found was not related to the clinical presence of AI. Further research is necessary to examine larger number of patients and genes related to AI.

## Figures and Tables

**Table 1:** Primers sequence (forward and reverse) of exons of the amelogenin gene and the PCR conditions.

Exon	Primer sequence 5′-3′	Fragment size (bp)	AT (°C)
2	F: AGATTATGTGTGTTTTATGGAGCA	233	62°C
	R: CCCTAATTTCACCAACTATGAGC		

3	F: TCCTTTAATGTGAACAATTGCAT	250	57°C
	R: TCTGGGATAAAGAATCAACACA		

4-5	F: AATGAATCTCTTTAACTCCCCATAA	367	60°C
	R: TCCCATTAATGTCTGCATGTG		

6	F: CCATAATGGCAAAGAAAACAC	599	59°C
	R: TGGTTGTCGGAGACCTTAGAA		

7	F: TGACAAAACTGAAGCCAGACAT	237	61°C
	R: TGCATTTTATTGTCTGCTAATGG		

F: forward; R: reverse; bp: base pairs; AT: annealing temperature.

**Table 2:** Electropherograms showing the silent mutation found (rs2106416) in sequences of mother, father, and brother of patient.

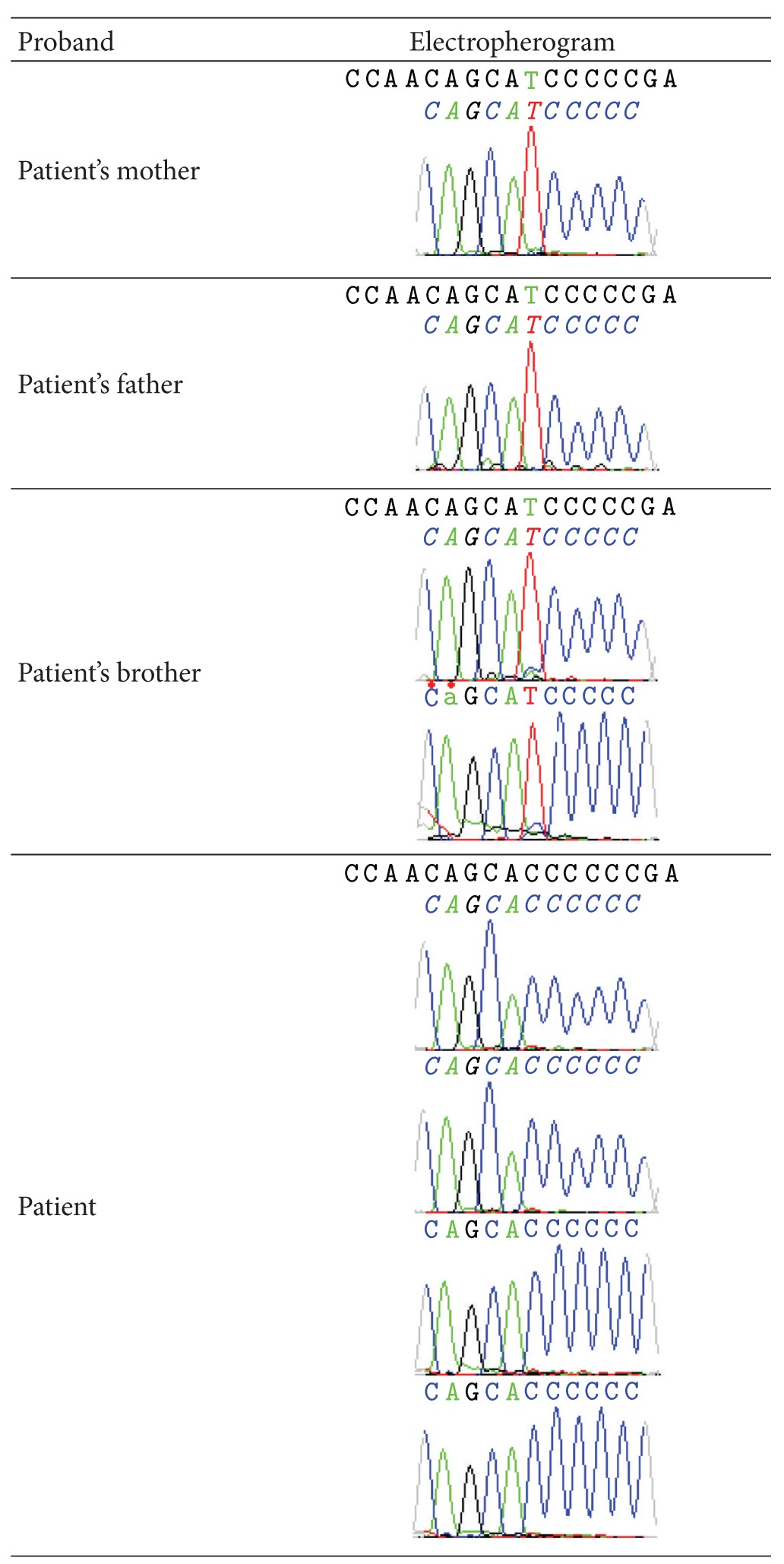

A: adenine; T: thymine; C: cytosine; G: guanine.

## References

[1] Witkop CJ (1988). Amelogenesis imperfecta, dentinogenesis imperfecta and dentin dysplasia revisited: problems in classification. *Journal of Oral Pathology*.

[2] Gisler V, Enkling N, Zix J, Kim K, Kellerhoff N-M, Mericske-Stern R (2010). A multidisciplinary approach to the functional and esthetic rehabilitation of amelogenesis imperfecta and open bite deformity: a case report. *Journal of Esthetic and Restorative Dentistry*.

[3] Stephanopoulos G, Garefalaki M-E, Lyroudia K (2005). Genes and related proteins involved in amelogenesis imperfecta. *Journal of Dental Research*.

[4] Crawford PJM, Aldred M, Bloch-Zupan A (2007). Amelogenesis imperfecta. *Orphanet Journal of Rare Diseases*.

[5] Kim J-W, Seymen F, Lin BP-J (2005). ENAM mutations in autosomal-dominant amelogenesis imperfecta. *Journal of Dental Research*.

[6] Pires Dos Santos AP, Cabral CM, Moliterno LF, Oliveira BH (2008). Amelogenesis imperfecta: report of a successful transitional treatment in the mixed dentition. *Journal of Dentistry for Children*.

[7] Wright JT, Torain M, Long K (2011). Amelogenesis imperfecta: genotype-phenotype studies in 71 families. *Cells Tissues Organs*.

[8] Doruk C, Ozturk F, Sari F, Turgut M (2011). Restoring function and aesthetics in a class ii division 1 patient with amelogenesis imperfecta: a clinical report. *European Journal of Dentistry*.

[9] Berglund EC, Kiialainen A, Syvänen A-C (2011). Next-generation sequencing technologies and applications for human genetic history and forensics. *Investigative Genetics*.

[10] Karger BL, Guttman A (2009). DNA sequencing by CE. *Electrophoresis*.

[11] Milos PM (2009). Emergence of single-molecule sequencing and potential for molecular diagnostic applications. *Expert Review of Molecular Diagnostics*.

[12] Bocchini CA (2011). 300391 Amelogenin; AMELX. *OMIM—Online Mendelian Inheritance in Man*.

[13] European Bioinformatics Institute WTSI Ensembl. Ensembl Project Human (Homo sapiens). http://www.ensembl.org/Homo_sapiens.

[14] Moretti ABS, Sakai VT, Oliveira TM (2007). Oral management of a child with mixed dentition affected by amelogenesis imperfecta. *Journal of Dentistry for Children*.

[15] Lindemeyer RG, Gibson CW, Wright TJ (2010). Amelogenesis imperfecta due to a mutation of the enamelin gene: clinical case with genotype-phenotype correlations. *Pediatric Dentistry*.

[16] Hart PS, Aldred MJ, Crawford PJM, Wright NJ, Hart TC, Wright JT (2002). Amelogenesis imperfecta phenotype-genotype correlations with two amelogenin gene mutations. *Archives of Oral Biology*.

